# Retrospective analysis of dengue-related fatalities in Nepal, 2022

**DOI:** 10.1371/journal.pgph.0004694

**Published:** 2025-06-02

**Authors:** Shashi Kandel, Gokarna Dahal, Chuman Lal Das, Susmita Thapa, Ajit Kumar Karna, Ashna Parajuli, Riju Aryal, Khin PaPa Naing, Nancy Lama, Bandana Pandey, Prabesh Ghimire, Rudra Prasad Marasini

**Affiliations:** 1 Department of Health Services, Epidemiology and Disease Control Division, Ministry of Health and Population, Kathmandu, Nepal; 2 Ministry of Health and Population, Kathmandu, Nepal; 3 Nepal One Health Institute, Kathmandu, Nepal; 4 World Health Organization, Kathmandu, Nepal; Keele University, UNITED KINGDOM OF GREAT BRITAIN AND NORTHERN IRELAND

## Abstract

Dengue is a mosquito-borne acute febrile illness, also known as break bone fever, and is a major public health problem in the tropics and subtropics worldwide. Understanding the factors that contribute to dengue-related mortalities is crucial for decision-making and implementing effective strategies for prompt patient care. This retrospective analysis aimed to understand the clinical characteristics as well as associated infections and co-morbidities related to dengue fatalities in Nepal. Additionally, this insight aids in developing targeted public health interventions to save lives, enhancing disease surveillance systems, and fostering community awareness about dengue prevention. We conducted a retrospective study of the dengue-related deaths in Nepal reported to the Epidemiology and Disease Control Division between 01 January and 30 November 2022 through early warning and reporting system. Medical records of 88 patients who died from dengue were collected and reviewed from 23 hospitals of Nepal. Among 88 deaths that were reviewed, 47 (53.4%) were males and 41 (46.6%) were females. Of all the death cases reviewed, 26% experienced septic shock, 23% had multiple organ dysfunction syndromes, 20% had a cardiopulmonary arrest, 15% had acute respiratory distress syndrome, and 5% had severe gastro-intestinal bleeding, before the death. Fatality from severe dengue were in 46 cases (52%), from dengue associated with other diseases were in 23 cases (26%), and from dengue associated with co-morbidity complications were in 19 cases 22%. Dengue-related mortality in Nepal disproportionately affected older adults with underlying health conditions and co-infections. Late presentation and rapid clinical deterioration were common. Strengthening early diagnosis, timely referral, and clinical management, particularly for high-risk groups is essential. Public awareness of dengue warning signs and prompt healthcare-seeking behavior should be a key component of dengue control strategies.

## Introduction

Dengue fever is an infectious disease caused by four antigenically distinct but related dengue virus serotypes (DENV-1, -2, -3, and -4) and the virus is spread to humans via the bites of infected *Aedes* mosquitoes [1]. It is one of the most extensively spread mosquito-borne diseases; endemic in more than 100 countries [2]. As many as 3.83 billion people, or 53% of the world’s population live in areas that are suitable for dengue transmission, with the vast majority being in Asia [3] About half of the world’s population is now at risk of dengue with an estimated 100–400 million infections occurring each year [4]. The World Health Organization 2009 has classified dengue fever patients into three groups; dengue without warning signs, dengue with warning signs, and severe dengue. Clinical manifestation of severe dengue clinically presents severe bleeding, multiple organ involvement, and widespread plasma leakage from the body. Most dengue deaths are associated with severe dengue [5].

Nepal has a historical record of all four dengue serotypes circulating, but in 2022, DENV-1 and DENV-3 were the most prevalent serotypes, with no evidence of DENV-4 to date. In the same year, Nepal reported 54,784 dengue patients and 88 deaths, marking the highest number ever recorded in the country [6]. Although fatality due to dengue increased over the years globally, the case fatality ratio remained between 0.03% and 0.93% from 2018 to 2022 [7].

Dengue appeared as a new disease in Nepal in 2004 from a Japanese traveler with yearly sporadic cases afterward [8]. It has become a major disease of concern in Nepal due to the detection of infection throughout the year, with cases recorded from all 77 districts nationwide. Dengue outbreaks in Nepal have been reported throughout all seasons- pre-monsoon, monsoon, and post-monsoon, even though the post-monsoon timeframe is thought to be a high transmission season for dengue. In 2019, the country recorded 17,992 dengue patients and six deaths, whereas in 2022 (as of 30 November), 53,951 dengue patients and 88 deaths were reported from all 77 districts [6]^.^ Dengue fever has become a recurrent and growing threat to public health in Nepal [8]. The spread of dengue fever in terms of morbidity, fatality, and geographical distribution has made it clear that immediate action is required to reduce dengue-related fatalities [9].

Recognizing the public health concern of dengue, the Epidemiology and Disease Control Division (EDCD) of the Department of Health Services/ Ministry of Health and Population conducted a review of all dengue-related deaths reported from around the country with the assistance of the World Health Organization (WHO) to better understand the clinical characteristics as well as other contributing factors and co-morbidities. A better understanding of the role of underlying comorbidities in the development of severe outcomes would allow for better targeting clinical management to ensure prompt, and aggressive supportive therapy for those at high-risk, and thus reduce dengue-related fatality. This is a retrospective analysis of clinical data on dengue-related deaths reported to the EDCD through its Early Warning and Reporting System (EWARS) between 01 January and November 30, 2022.

## Materials and methods

### Data sources and data collection

Between January and November 2022, a total of 53,951 dengue patients were reported nationwide in Nepal, with Bagmati province reporting the largest number of cases (41,947), followed by Lumbini province (4,975) and Koshi province (2,198). Eighty-eight dengue deaths were reported in EWARS in the same period. EDCD conducted follow-up visits to dengue death reporting hospitals to verify reported deaths. During these visits, EDCD officials obtained photocopies of existing hospital records pertaining to diagnosis, investigation, and treatment. This process involved no direct interaction with patients or their families, and no additional data were collected from treating physicians beyond what was already recorded in hospital databases. The clinical data for the 88 death cases reported from 23 hospitals (both government and private) were fully anonymized by EDCD and were made available for review and analysis. This was performed from 1^st^ of November through 17^th^ of December, 2022. Patient information on demographics, clinical characteristics, clinical management, and laboratory findings (S1 Data) were collected using a structured proforma developed by public health and infectious disease specialists and approved by EDCD/ DoHS.

### Ethical consideration

This study utilized existing data gathered by the National Dengue Control Program through the EDCD, with contributions from hospital-reported cases. The data was anonymized by EDCD, ensuring confidentiality, and shared with the study team devoid of any identifying information. Authors associated with the National Dengue Control Program had access to the data prior to analysis, but personal identifiers were meticulously removed by EDCD to protect patient anonymity. Since this research relied solely on secondary data and did not involve direct contact with human subjects, ethical approval was not sought.

### Data management and data analysis

Based on a review of patient records, the type of dengue present at the time of admission was categorized into three groups dengue without warning signs; dengue with warning signs (abdominal pain, persistent vomiting, rapid breathing, bleeding gums or fatigue, restlessness, enlarged liver, and signs of clinical fluid accumulation); and severe dengue (severe plasma leakage, fluid accumulation with respiratory distress, severe bleeding, or organ dysfunction) [10]. Length of hospital stay was categorized into three groups: less than one day; one to three days, or more than three days. All patients who tested positive for dengue using the Rapid Diagnostic Test (RDT) kit were labeled based on the presence of NS1 antigen, IgM, or IgG-positive antibodies. Travel history, previous dengue infections, and family history with dengue were also investigated; however, the availability of such data was minimal. Antibiotic usage and IV fluid types were also assessed and analyzed. In addition, data on the patients who received hemodialysis, as well as invasive and non-invasive ventilation were examined. The study team also looked at the results of the total leucocyte count, platelet count, serum creatinine, liver enzymes, hematocrit, USG, and chest x-ray. Types of blood products that were transfused were analyzed. Co-infections such as scrub typhus and leptospirosis were validated using serological test reports; pulmonary tuberculosis was validated using radiological findings and a sputum profile available in the patient record; and the presence of COVID-19 was validated using RT-PCR records of patients. Descriptive statistics were used to present the findings of this review.

## Results

### Demographic profile

**Fig 1:**
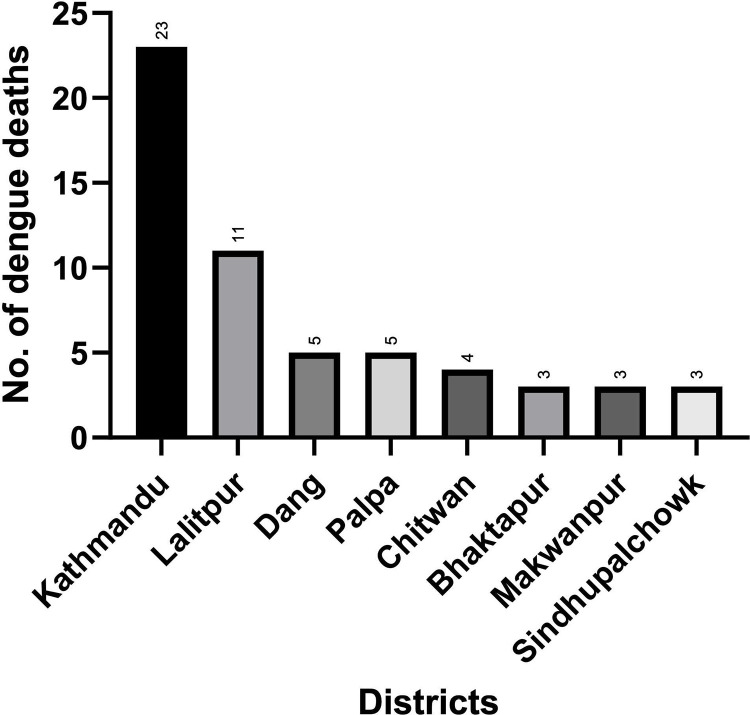
Distribution of dengue-related deaths by district (*n* = 88).

The demographics of all fatal cases are illustrated in Supporting Table 1. Among 88 deaths that were reviewed, 47 (53.4%) were males and 41 (46.6%) were females. The age range was between 3–90 years with a median age of 58 years. Adults over 15 years old comprised 96.6% (85 patients), with 43 deaths recorded in 15–49 years age (16 – 59 years range, median 42 years) and 42 deaths in age 60 years and above (60–90 years range, median 72 years). Three deaths occurred in 3–10 years age range, with 4 years median age. Half of the death cases (53.4%) were males. Brahmin/ Chhettri and Janajati were equal in number in the death records, 39.77% (35) and 40.91% (36) respectively, while others were – Dalit 2.27% (2), Madheshi 5.68% (5), Muslim 1.14% (1), and others 10.23% (9). It was observed that the case fatality was higher in the above 55 years age group with co-morbid conditions, other infections, severity of dengue, and associated complications. Death cases reported from 23 hospitals (13 hospitals within Kathmandu Valley and 10 hospitals outside the Kathmandu Valley) were reviewed. The majority of the deaths were reported from Bagmati province (60.2%, 53 deaths) followed by Lumbini province (19.3%, 17 deaths). Fig 1 displays the distribution of dengue-related deaths by districts. Most cases were reported from Katmandu (23), Lalitpur (11), Palpa (5) and Dang (5) districts. The case fatality rates for Kathmandu and Lalitpur were 0.16% and 0.11%, respectively.

**Table 1:** Demographic profile of fatal dengue cases (*n* = 88).

Demographics	1-4 Years (%)	5-14 Years (%)	15-25 Years (%)	25-34 Years (%)	35-54 Years (%)	55-74 Years (%)	75 + Years (%)
Cases (per overall)	2 (2.3)	1 (1.1)	4 (4.5)	10 (11.4)	22 (25)	31 (35.2)	18 (20.5)
Gender							
Male	0	0	2 (50)	4 (40)	8 (36.4)	21 (67.7)	12 (66.7)
Female	2 (100)	1 (100)	2 (50)	6 (60)	14 (63.6)	10 (32.3)	6 (33.3)
Ethnicity							
Brahmin/ Chettri	0	0	2 (50)	2 (18.2)	8 (36.4)	14 (45.2)	9 (50)
Dalit	0	0	0	0	0	1 (3.2)	1 (5.6)
Janajati	2 (100)	1 (100)	0	5 (45.5)	8 (36.4)	12 (38.7)	8 (44.4)
Madheshi	0	0	1 (25)	0	1 (4.5)	3 (9.7)	0
Muslim	0	0	0	2 (18.2)	0	0	0
Others	0	0	1 (25)	2 (18.2)	5 (22.7)	1 (3.2)	0

### Clinical profile

Of the 88 death cases that were reviewed, the majority of the cases (90.9%) were admitted to the hospital through the emergency department, and only a few cases (9.1%) through the outpatient department. Among them, nearly half of the patients (40.9%) were referred from other hospitals for further management, while the remaining 59.1% had been directly admitted to the study hospital. Nearly half of the cases (47.2%) were hospitalized within 5 days of the onset of symptoms. The majority (43.2%, 38 patients) of the deceased patients stayed in the hospital for 1–3 days, followed by 36.4% (32 patients) for more than 4 days and 20.4% (18 patients) for less than one day. Similarly, majority (65.9%, 58 patients) stayed in private tertiary hospital, followed by 19.3% (17 patients) in public tertiary hospital, 12.5% (11 patients) in public district level (secondary) of hospital, and 2.3% (2 patients) in private secondary level hospital.

### Diagnosis

Table 2 illustrates the findings of dengue diagnostic tests based on RDT. All 88 cases had a rapid diagnostic test (RDT) as a confirmatory test, and 43 of them tested positive for NS1 antigen, 29 tested positives for IgM, and seven tested positives for both NS1 and IgM.

**Table 2:** Findings of dengue diagnostic tests based on RDT (*n* = 88).

Cases	NS1 Ag	IgM	IgG	NS1 Ag + IgM	NS1 Ag + IgG	NS1 Ag + IgM + IgG	IgM + IgG	All Negative
Number of cases	43	29	3	7	3	1	2	0

### Dengue classification at the time of hospital admission

**Fig 2:**
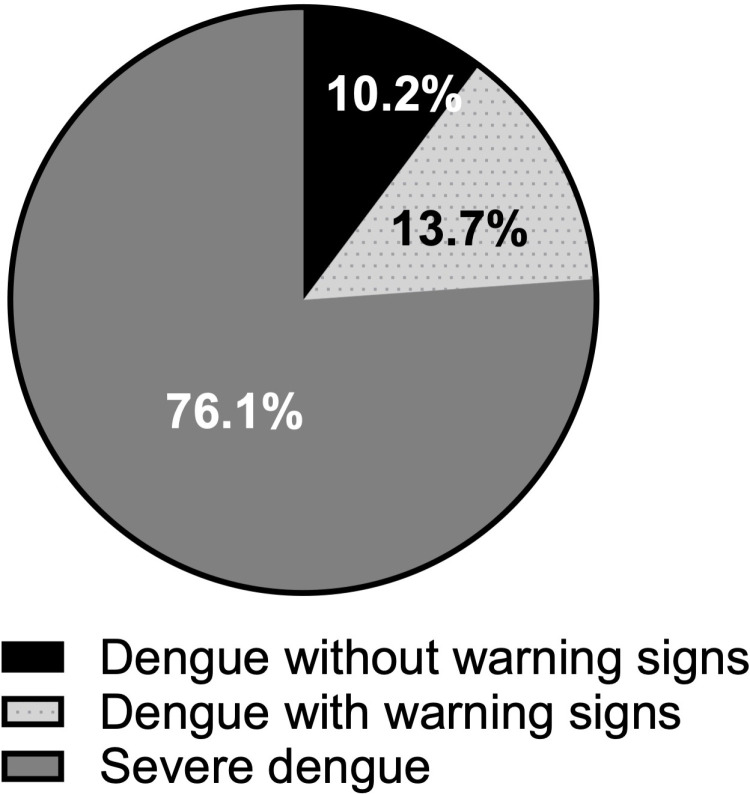
Distribution of dengue cases based on WHO classification at the time of hospital admission.

Fig 2 displays the distribution of dengue cases based on WHO classification at the time of hospital admission. At the time of admission, nine of the 88 dengue cases had no warning signs, 12 had warning signs, and 67 had severe dengue. The nine patients who arrived at the hospital with dengue without warning signs were admitted for observation for associated co-infections (three cases of pneumonia and one case of urinary tract infection and four cases of co-morbidity, including one case of chronic hyponatremia in a 90-year-old patient), and others who had persistent dengue symptoms that did not improve with oral medications. As the illness progressed, all patients experienced at least one symptom of severe dengue.

### Common signs and symptoms at admission

Table 3 shows the common signs and symptoms of dengue patients at the time of admission. The most common symptoms reported were fever (74 cases, 84.1%) respiratory distress (49 cases, 55.7%), lethargy (33 cases, 37.5%), myalgia (26 cases, 29.5%), vomiting (26 cases, 29.5%), headache (25 cases, 28.4%), abdominal pain (23 cases, 26.1%), and severe bleeding (18 cases, 20.5%). Less than ten percent of cases had also experienced altered sensorium, retro-orbital pain, rash, cough, loose stool, and seizure.

**Table 3:** Common signs and symptoms of dengue patients at the time of hospitalization (*n* = 88).

Common signs and symptoms	Count of patients	Percent
Fever	74	84.1
Respiratory distress	49	55.7
Lethargy	33	37.5
Myalgia/ arthralgia	26	29.5
Vomiting	26	29.5
Headache	25	28.4
Abdominal pain	23	26.1
Bleeding	18	20.5
Altered mental status	7	8.0
Retro-orbital pain	6	6.8
Rash	4	4.5
Cough	6	6.8
Loose stool	5	5.7
Seizure	2	2.3
Tourniquet test positive (10 or more petechiae/ sq inch)	2	2.3

### Associated infections

Table 4 shows the associated infections of dengue patients. Of total dengue deaths, 32 cases had one or more associated co-infections. Among them, 68.8% had pneumonia, followed by scrub typhus cases (21.8%) and one case each with pulmonary tuberculosis (in the initial phase of antitubercular treatment), leptospirosis, COVID-19, and enteric fever.

**Table 4:** Associated infections of dengue patients (*n* = 32).

Co-infections	Count of patients	Percent
Pneumonia	22	68.75
Scrub typhus	7	21.88
COVID-19	1	3.12
Enteric fever	1	3.12
Leptospirosis	1	3.12
Pulmonary TB	1	3.12
Others	1	3.12

Furthermore, among the co-morbid cases (Table 5), hypertension accounted for 56.1% (32 cases), followed by 38.6% diabetes mellitus (22 cases) and 29.8% pulmonary disease (17 cases), cases, 29.82% pulmonary disease (17 cases, 10.52% ischemic disease (6 cases), 8.77% alcoholic liver disease (5 cases), 8.77% chronic kidney disease (5 cases), 8.77% cerebral vascular disease (5 cases), and 36.84% represented others (21 cases).

**Table 5:** Presence of co-morbid among children, adults, and elderlies upon hospital admission (*n* = 57).

	No. of patients (%)	Children (< 15 years) *n* (%)	Adults (15–59 years) *n* (%)	Elderlies (≥ 60 years) *n* (%)	P value
Presence of co-morbid	57 (64.8%)	0	36 (63.2)	21 (36.8)	
Co-morbidities:					
*Hypertension*	32 (36.4)	0	11 (34.4)	21 (65.6)	0.02*
*Diabetes mellitus*	22 (25)	0	7 (31.8)	15 (68.2)	0.07
*Pulmonary disease*	17 (19.3)	0	4 (23.5)	13 (76.5)	0.02*
*Ischemic disease*	6 (6.8)	0	1 (16.7)	5 (83.3)	0.19
*Alcoholic liver disease*	*5 (5.7)*	0	1 (0.2)	4 (0.8)	0.33
*Chronic kidney disease*	*5 (5.7)*	0	3 (60)	2 (40)	0.82
*Cerebral vascular disease*	*5 (5.7)*	0	1 (20)	4 (80)	0.32
*Others*	*21 (23.9)*	0	7 (33.3)	14 (66.7)	0.02*

### Clinical management

Prior to presenting to the hospital, a total of 18 patients were taking medications for dengue symptoms, but there was no clear information about whether the medications taken were prescribed in the previous hospital where they were referred from or taken even prior to admission in previous hospitals. Seven (8%) of the patients were taking anti-platelet medication for their underlying conditions, but only one of these patients had severe bleeding and four patients had thrombocytopenia. It is important to further investigate the link between anti-platelet drug use and case fatality. The duration of hospital stays ranged from 1-3 days (median 2 days) for all 88 patients, with 1–3 days for 38 patients (43.2%), 4 days or more for 32 patients (36.4%), and less than 24 hours for 18 patients (20.5%). Out of the 88 deaths that were reviewed, 87 (98.9%) cases received intravenous (IV) fluid therapy. Of these, 84 cases (96.6%) received crystalloid fluid, and three cases (3.4%) received both crystalloid and colloid. However, the volume of IV fluid received was not well documented. A total of 76 (86.4%) patients were treated with IV antibiotics for secondary infections. IV antibiotics were administered to 76 (86.4%) cases in total for secondary infections. Blood transfusions were received in 24 cases (27.3%), of which 11.4% received platelet-rich plasma and 10.2% received a combination of blood products.

Of all patients who died of dengue, 13.6% (12 cases) had received hemodialysis for acute kidney injury developed during their hospital stay, with 4.5% having chronic renal illness since prior to admission and receiving maintenance hemodialysis. In critical condition, 43 patients were kept on mechanical ventilation for control ventilation, while 11 were kept on a non-invasive ventilation approach. Twelve patients (13.6%) had opted for “Do not resuscitate” (DNR)/ “Do not ventilate” (DNV) while the remaining 22 patients had not shown signs of return of spontaneous circulation (ROSC) after initial resuscitation. The lowest platelet count documented in a medical record was 8000 per microliter of blood. During hospitalization, three patients (3.6%) had a platelet count of less than 10,000 cells per microliter of blood and all three were given blood transfusions. At the time of initial presentation, 36 (42.9%) of patients (out of 84 cases with information on leucocytes) had leukocytosis and 17(20.2%) had leucopenia. Increased hematocrit levels were observed in 17 (21.3%) of the cases (out of 80 cases with information on hematocrit levels).

A significant increase in liver enzymes was seen, with SGPT raised in 53.9% of patients (n = 76) and SGOT raised in 65.8%. Other co-infections with dengue such as scrub typhus, (4 out of a total 7 scrub typhus cases) and one leptospirosis may have contributed to the elevated liver enzymes. At the time of hospital admission, 34 (44.2%) cases (among 77 cases for which information on creatinine levels was available) had raised serum creatinine levels. Gall bladder wall thickening was present in seven out of the 73 patients who had an abdominal ultrasound. Additionally, 19 patients (out of 79 cases) displayed signs of fluid accumulation such as pleural effusion, ascites, pericardial effusion, and dependent edema.

### Cause of death

The clinical course of dengue is dynamic and might change in ways that were not expected. It is still difficult to determine the exact cause of death in each case because some patients’ deaths may have been caused by multiple factors. It was observed that the mortality was higher in the above 55 years age group with co-morbid conditions, other infections, severity of dengue, and associated complications. Six of the 36 cases that were referred in, died within a few hours of arrival at the hospital. Out of 47 severe dengue cases, 22 (46.8%) died within three days of hospital admission which accounts for the highest number of deaths to hospital stay. Among 23 deaths from severe dengue associated with co-infection, 14 (60.8%) deaths were among those who had a hospital stay of more than or equal to four days.

Table 6 shows the contributory causes of death in dengue patients. Out of all death cases reviewed, 26% had experienced septic shock, 23% had multiple organ dysfunction syndromes, 20% had cardiopulmonary arrest, 15% had acute respiratory distress syndrome, and 5% had severe gastrointestinal bleeding, before the death. Less than 5% of individuals who died from dengue had acute renal injury, pulmonary edema, or disseminated intravascular coagulation (DIC). Mortality from severe dengue was 52.3 percent (46 deaths), from severe dengue associated with other infections, was 26.1 percent (23 deaths), and from severe dengue associated with co-morbidity complications was 21.6 percent (19 deaths).

**Table 6:** Contributory causes of death (*n* = 88).

Contributory Causes	Male (%)	Female (%)	Total
Septic shock	11 (47.8%)	12 (52.2%)	23
Multi-organ dysfunction syndrome (MODS)	13 (61.9%)	8 (38.1%)	21
Cardiac pulmonary arrest	9 (50%)	9 (50%)	18
Acute respiratory distress syndrome (ARDS)	9 (64.3%)	5 (35.7%)	14
Severe bleeding	1 (20%)	4 (80%)	5
Pulmonary edema	1 (50%)	1 (50%)	2
Acute kidney injury (AKI)	1 (50%)	1 (50%)	2
Myocarditis	1 (50%)	1 (50%)	2
Disseminated intravascular coagulation (DIC)	1 (100%)	0 (0%)	1

## Discussion

Dengue, a preventable cause of mortality, [11] can be prevented by health promotion activities and vector control. Nevertheless, in instances of infection, the risk of death can be significantly reduced through prompt, accurate diagnosis and effective treatment strategies. Therefore, awareness of health care professionals is as fundamental as ready accessibility to health care services [12]. The steady increase in the number of dengue patients in the past few years can be attributed to rapid urbanization and unplanned construction activities [13].

In our study, we found that individuals over the age of 55 constituted half of the fatal cases observed, which is also supported by Lee et al. 2022 in which the majority (90%) of death cases were ≥ 65 years of age [14]. Similarly, in another study in Brazil, a significantly higher chance of death (two-fold greater chance) from severe dengue was associated with an age ≥ 50 years [12]. Likewise, in our study, more than half of the cases were concentrated in Bagmati province, particularly in Kathmandu. This high incidence could be attributed to issues with the mismanagement of waste disposal systems, which coincided with a peak in the same year as a result of poorly planned urban areas [15]. Another contributing factor could be the presence of a tertiary care center in Kathmandu and the resulting higher case referral rate.

Almost all of the cases were admitted through the emergency department which tends to suggest that people visit hospitals only when their symptoms worsen. This behavior might be driven by the perception of illness severity and a preference for immediate emergency care. Similarly, nearly half of the cases in our study were referred cases, and a significant majority of these referred patients chose to seek care at the emergency department rather than the outpatient department (OPD). This preference for the emergency department can be attributed to the fact that cases are typically referred when their symptoms worsen, necessitating more immediate and intensive medical attention.

Nearly half of the cases were hospitalized within 5 days of the onset of the symptoms which is also supported by Abello et al. 2016 which shows that in the Philippines, the majority of cases were admitted during the period between the third and fifth day from the onset of the disease [16]. The timing of hospital admission has been associated with the severity of the disease. Typically, non-severe cases are admitted to the hospital on the third or fourth day since the onset of diseases, whereas more severe cases tend to be admitted later [17]. According to a study by Moraes et al. 2013, there is a higher probability of death among patients who had their case notified after four days from the onset of symptoms compared with those whose cases were notified earlier [12].

In our study most common symptoms reported were fever followed by respiratory distress, lethargy, and myalgia which is also supported by Acharya et al. 2018 which shows that common symptoms reported were fever, myalgia, and headache respectively and a similar study shows that 45.5% of cases suffered from ARDS [18]. A study by Chang et al. 2018 also shows that almost all the cases were associated with fever, 51.4% with myalgia, 49.7% with headache, and 48.9% with a loss of appetite [19].

Duration of dengue viremia has been demonstrated to correlate with the duration of the fever, meaning that viremia persisted while the patient was febrile and subsided as the fever subsided [20]. It may be postulated that prolonged fever may equate to prolonged dengue viremia, although there is no current study to prove this theory [21]. Diagnosing secondary hemophagocytic lymphohistiocytosis (HLH) during a dengue fever outbreak can be difficult. However, it’s important to consider HLH as a possibility in patients who have an ongoing fever, a decrease in all types of blood cells (pancytopenia), and problems affecting multiple organs [22].

In our study approximately one-fifth of the cases had severe bleeding and few cases had severe GI bleeding before death. Similar findings have been found in a study in South India by Acharya et al. 2018 in which the presence of bleeding tendencies including gastrointestinal bleeding and hematuria were identified as the clinical markers of risk of death [18]. Likewise, the study in Brazil conducted by Pinto et al. 2016 highlighted that gastrointestinal bleeding emerged as the clinical sign most significantly linked to a heightened risk of death [23]. Likewise, in our study few cases had a platelet count of fewer than 10,000 cells per microliter of blood, similar findings have been shown by Daniel et al. 2005 in which 8.6% had a platelet count of less than 10,000 cells per microliter of blood [24]. The cause of thrombocytopenia during dengue infection is still unknown but may be related to severe suppression of bone marrow populations including megakaryocytes, the progenitors of platelets [13].

Only a few patients were under antiplatelet therapy for their underlying conditions, in which one of them experienced severe bleeding. In contrast, a study done in Indonesia shows that none of the Dengue Hemorrhagic Fever (DHF) patients under antiplatelet therapy (clopidogrel 75 mg) experienced severe bleeding, except for minor cutaneous bleeding despite of continuation of antiplatelet therapy during the infection period [25]. These differences might be because of platelets count >20,000/mm^3^. Likewise, another study conducted in Singapore in 2020 shows that there was also no statistically significant difference in terms of case fatality between patients who continued antiplatelet therapy and patients who discontinued [26]. The decision to stop antiplatelet agents and anticoagulants in patients with dengue fever depends on a complex risk-benefit assessment of these therapies [27]. Thus, future studies are required to evaluate the safety profile on the continuation and or discontinuation of antiplatelet therapy for adult patients with dengue with different cardiovascular risk profiles to allow for individualized and personalized care [26].

In our study, a significant increase in liver enzymes was seen and is similar to findings from other studies [18,24,28,29]. The pathophysiology of liver involvement is not clear. Liver damage in dengue may be related to direct cytopathic effects and host immune response, as well as hypoxic damage secondary to microvascular leakage and/or shock. Frequently used medications for symptomatic relief, such as acetaminophen, may also contribute to liver injury [30].

Most of the cases in this analysis experienced septic shock, followed by MODS, cardiac arrest, ARDS, and GI bleeding respectively, which is also supported by Ong et al. 2007 in which major causes of death were multi-organ failure with adult respiratory distress syndrome, pneumonia and/or septicemia, intracerebral hemorrhage [31] and the study by Kaur et al. 2020 [13] conducted in North India in which major cause of death were shock, MODS, dengue encephalopathy, ARDS and intracranial hemorrhage respectively. In Thailand in 2017, among 874 adult patients with community-acquired sepsis enrolled in hospital, 126 patients were positive for dengue which shows that sepsis is also one of the major predictors of death in dengue cases [13].

In our study out of 47 severe dengue cases, 22 (46.8%) died within three days of hospital admission which accounts for the highest number of deaths with hospital stays which is also supported by Ong et al. 2007 in which all dengue-death cases had rapid progressive clinical deterioration at an average of day 4 of fever noted as the date of admission [31]. Similarly, a study in Taiwan by Lee et al. 2022 shows that more than half of the cases died within 1 week of hospitalization [14].

In our study more than half of the cases had comorbid conditions, major accounting for hypertension, followed by DM and pulmonary disease which is supported by a study in Taiwan in 2018 by Chang et al. 2018 which shows that DHF had a significantly stronger association with some chronic diseases, such as hypertension, diabetes, renal diseases, hepatic diseases, cardiovascular diseases, thyroid disease and arthritis [19]. Likewise in another study by Lee et al. 2022, the major underlying medical illnesses were hypertension (53.3%), diabetes mellitus (50%), and chronic kidney disease (33.3%) [14]. Likewise in other studies, nearly half of the cases (42.8%) had pre-existing co-morbidities including diabetes, hypertension, and hyperthyroidism [31]. All of the findings conclude that preexisting co-morbidities further deteriorate the conditions in dengue.

### Limitations of the study

- This review was constrained by limited data on the time interval between the onset of symptoms and the dengue test, laboratory results, and vitals recorded during hospital admission, travel history, details on dengue re-infection, and history of dengue contacts in the family.
- This study was based on a review of hospital patient records, and verbal autopsies were not included in the study framework.

## Conclusion

This study highlights key factors contributing to dengue-related mortality in Nepal, notably among individuals over 55 years of age and those with pre-existing comorbidities or co-infections. Most patients were admitted through emergency departments in critical condition and died within a few days of hospitalization. The findings underscore the urgent need to raise community awareness about dengue warning signs and promote early medical intervention to reduce fatal outcome.

## Recommendations

This review on the fatal cases of the dengue patients has identified several areas that might need attention for reducing the incidence of any dengue-related fatalities in the future:

- It might be beneficial to closely monitor patients with existing health issues and those who have previously experienced dengue, as this approach may greatly reduce the likelihood of complications arising from dengue.
- Investigating patients with abnormal liver function tests (LFTs), ongoing fever, or those at high risk for co-infections such as scrub typhus, leptospirosis, enteric fever, tuberculosis, and the use of anti-tubercular medications might be beneficial.
- AKI, ARDS, MODS, and DIC can develop as complications of severe dengue (previously called expanded dengue shock syndrome/Dengue hemorrhagic fever) and may contribute to death, so prompt identification and management of such complications might reduce the case fatality related to dengue.
- The findings of this study indicate that individuals aged 55 years and over are more affected so it might be beneficial to give attention to this group taking into consideration the increased likelihood of complications arising from age-related susceptibilities and co-morbidities.
- Six patients (16.7%) of the patients that were referred, died within 24 hours, highlighting the importance of making an early decision to refer to a higher center.

Considering our findings, despite the availability of dengue prevention, control, and management guidelines, as well as a strong commitment to dengue control, dengue death cases have increased significantly, demonstrating the need for additional research on case fatality due to dengue to develop more effective and long-lasting interventions to reduce death due to dengue rates. Prevention of dengue disease is imperative to reduce morbidity and mortality. Diagnosing dengue is challenging because of its nonspecific clinical presentation. Strengthening health workers’ capacity for early diagnosis and clinical management at peripheral levels is critical for timely treatment and referral, and thus reducing dengue deaths.

## Supporting information

S1 Data An anonymized line list of 88 dengue-related fatal cases reported in Nepal, 2022. The dataset includes demographic, clinical, laboratory, and hospital management variables, compiled from 23 reporting hospitals.
(XLSX)
